# Converting *Access Microbiology* to an open research platform: community survey results

**DOI:** 10.1099/acmi.0.000272

**Published:** 2021-09-07

**Authors:** Alexandra M. Howat, Justin Clark

**Affiliations:** ^1^​ Microbiology Society, London, UK

**Keywords:** open science, open research platform, publishing, preprints, society publisher, Wellcome Trust

## Abstract

Following the Microbiology Society’s successful bid for a Learned Society Curation Award from the Wellcome Trust and Howard Hughes Medical Institute, the Society is converting our sound science, open access journal, *Access Microbiology*, to an open research platform. As part of this, we conducted a survey of our community to gauge current attitudes towards the platform and here we present some of these results. The majority of respondents (57 %) said they would always or sometimes want to remain anonymous on their peer review report, whilst 75 % of respondents said that as an author they would be happy to make the data underlying their research open. There was a clear desire for a range of research types that are often seen with sound science publications and rigorous research. An encouraging 94 % of respondents stated that the platform is somewhere they would consider publishing, demonstrating the enthusiasm in these respondents for a new publishing platform for their community. Given this data and that from our previous focus group research, the platform will launch as outlined in the original project proposal and adopt a transparent peer review model with an open data policy.

## Data Summary

The full survey dataset has been deposited in Figshare: https://doi.org/10.6084/m9.figshare.14696316.v1 [1]. Some data has been omitted or changed to preserve the anonymity of the respondents; the locations of the changes are indicated.

## Introduction

In July 2020, the Microbiology Society won a Learned Society Curation Award grant from the Wellcome Trust and Howard Hughes Medical Institute [2]. Our proposal was to convert our sound science, open access journal, *Access Microbiology*, into an open research platform. The journal currently operates a traditional single-blind publication model, with the published Version of Record the only version of the article that is made publicly available. Using our current technology, this project aims to turn the peer review process inside out, so that the entire lifecycle of the article is posted on the platform for all to see. This will include posting all versions of the article as preprints on the platform, and posting the peer reviews and the Editor’s decision alongside these, thereby increasing the transparency of the peer review process. The platform will also incorporate various manuscript review tools during the submission process, allowing authors to improve their article right from the very beginning of the peer review process.

In February 2021, we undertook research using focus groups to assess how our community felt about the platform, including the general model, the manuscript review tools that might be incorporated, and some of the key policies to be adopted [3]. Whilst we gained invaluable insight and feedback on many aspects of the platform through this method, there was a clear divide in how participants felt about the type of peer review model to be implemented (i.e. open or transparent) and the extent to which the data underlying results should be made open (open data). Given the opposing viewpoints being shared, and the small number of participants in the focus groups, we decided to obtain a wider sample of these important aspects of the platform from our community by conducting a survey. Here, we report on some of the main results and include recommendations.

## Methods

### Data collection

Between 14 April–14 May 2021 a link to the survey was shared via multiple avenues with the aim of gathering as diverse a sample as possible of the microbiology community and of key stakeholders. Methods included posting on Microbiology Society social media platforms (Twitter, LinkedIn), via other Society content (Microbe Post, monthly newsletter), inclusion within letters sent by the peer review system (Editorial Manager), direct emails to all corresponding authors who have published in *Access Microbiology,* and direct emails to Committee and Council members of the Society. Implicit consent to participate was given by respondents choosing to continue to take part in the survey after being informed at the beginning that the data collected would be published and that it would be anonymized. The full list of survey questions is provided in the Box at the end of the article.

### Data analysis

Data was checked for any possible identifying information, removed or edited, and deposited in Figshare: https://doi.org/10.6084/m9.figshare.14696316.v1. All responses were included in the analysis for each respective question, including those who did not complete all questions. Total number of responses (*n*) to each response type is included in all results.

## Results

Over the course of a month, we received 178 responses to the survey.

### Open versus transparent peer review

**Fig. 1.**
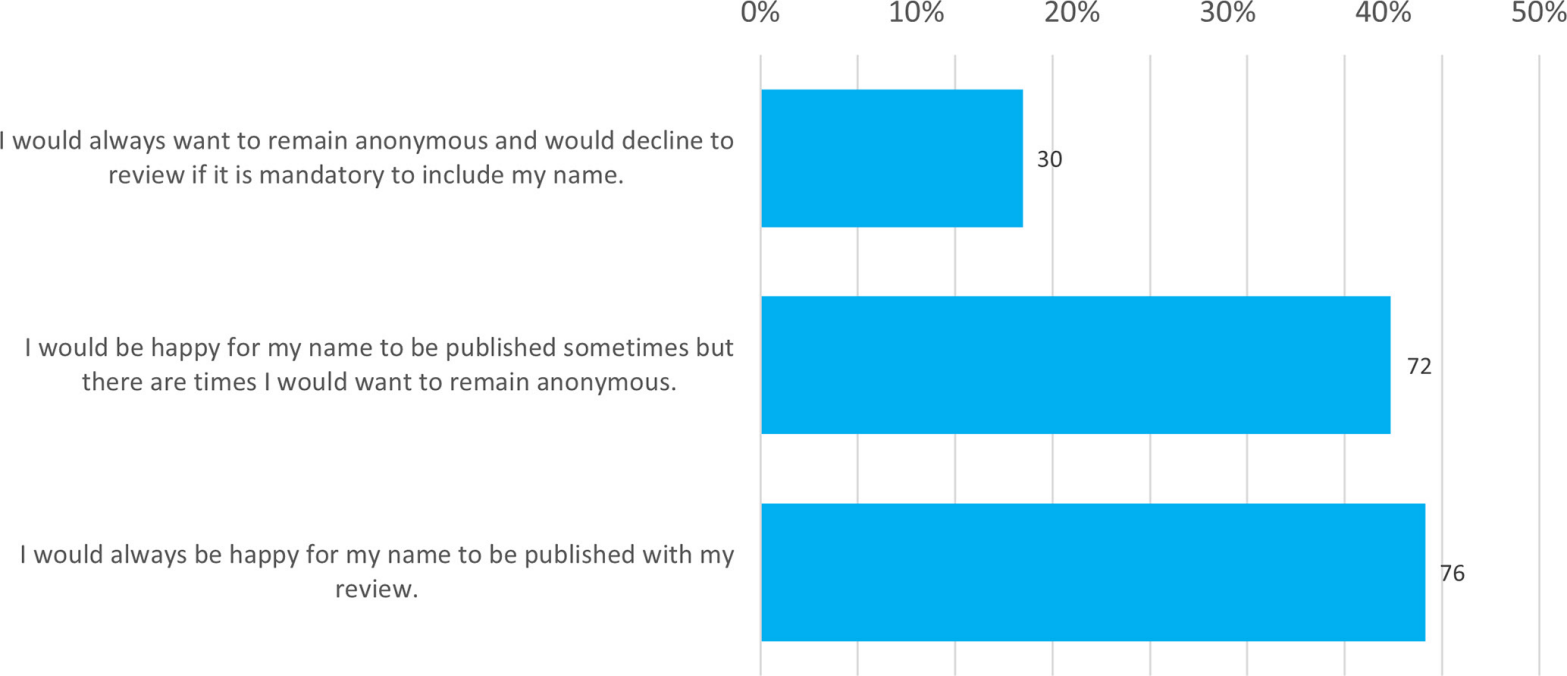
Willingness of respondents to include their name to their review. Answers shown were given in response to the question, ‘To increase transparency during the peer review process, the platform will publish peer review reports for each version of an article, preferably with the reviewer name(s) alongside. Knowing this, how would you feel about providing your name to your review?’ Total responses are indicated above each bar.

Whilst 43 % of respondents said they would always be happy to provide their name to their review, the majority (57 %, *n=*102) stated they would always (17 %, *n=*30) or sometimes (40 %, *n=*72) want to remain anonymous (Fig. 1).

**Fig. 2.**
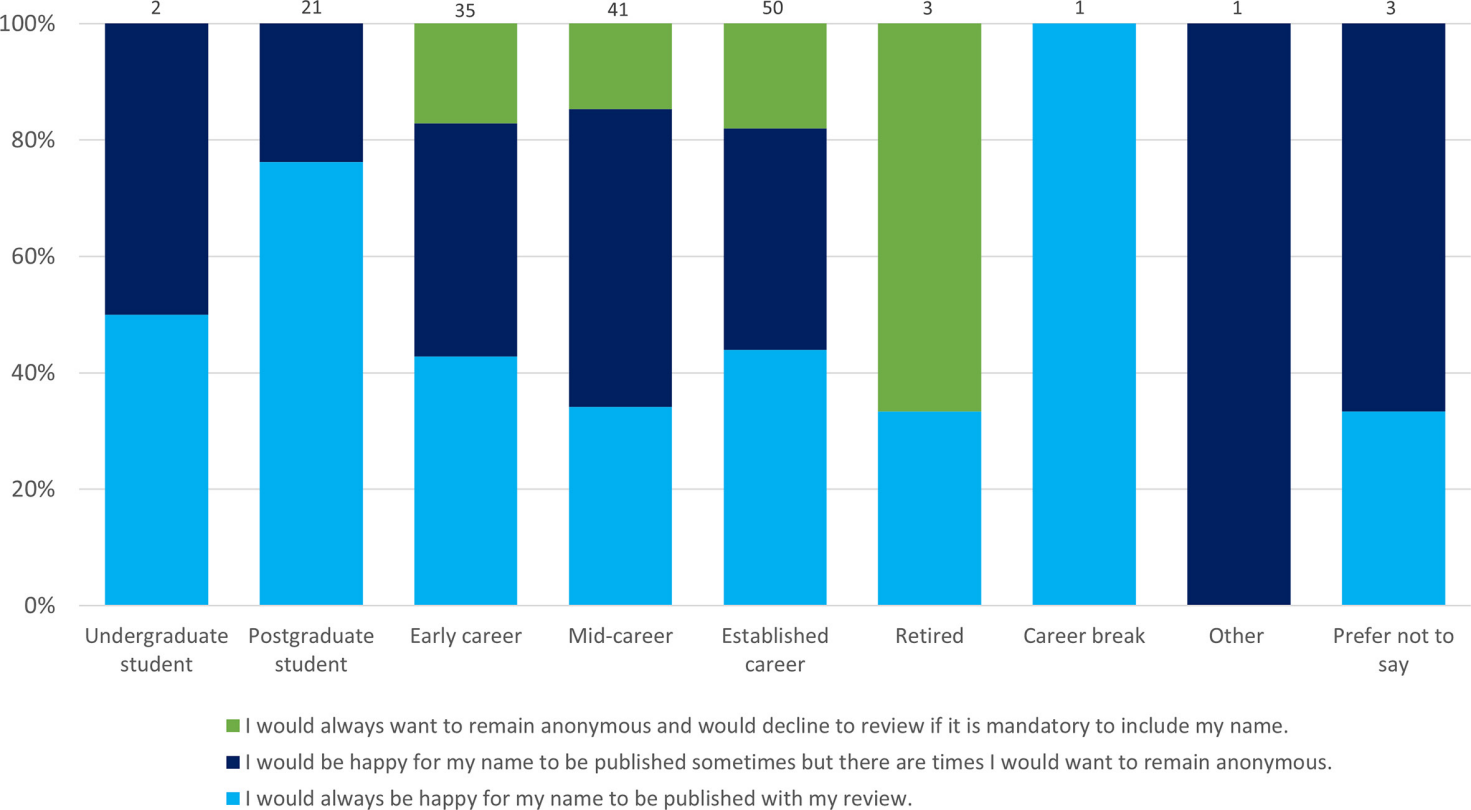
Willingness of respondents to provide their name to their review by career stage. Comparing responses to the questions ‘To increase transparency during the peer review process, the platform will publish peer review reports for each version of an article, preferably with the reviewer name(s) alongside. Knowing this, how would you feel about providing your name to your review?’ and ‘What career stage are you currently at?’. Total responses are indicated above each bar.

Slightly surprisingly, there was little difference in willingness to provide names to reviews between ‘Early career’ researchers compared to those in an ‘Established career’ (Fig. 2). An argument often made against open review is that it disproportionately impacts those in the earlier stages of their careers, as they may not wish to openly criticize more senior and well-established members in their field for fear of it impacting their career. These data suggest that the Early Career researchers surveyed do not necessarily feel more strongly towards remaining anonymous than the more established researchers. Also of interest was that ‘Postgraduate students’ (76 %) were the most likely of all groups to provide their name to their review. Whilst this might be a promising sign that attitudes are changing, it may also be due to the fact that many postgraduates are inexperienced in the publishing process, and in reality this view may change in practice.

When looking at why respondents indicated that they would sometimes or always wish to remain anonymous, the most frequently cited or alluded to reasons were: not wanting to risk their career progression by performing a critical review of a senior researcher; avoiding potential conflicts with the authors in the future; and that it is easier to provide critical reviews anonymously (see supporting data: https://doi.org/10.6084/m9.figshare.14696316.v1). Other reasons provided across the board were: a general wish to maintain anonymity; difficulty in remaining objective with friends or colleagues; fear of online or personal attacks; concern their review was of insufficient quality; and believing that open peer review does not provide good quality reviews.

### Open data

**Fig. 3.**
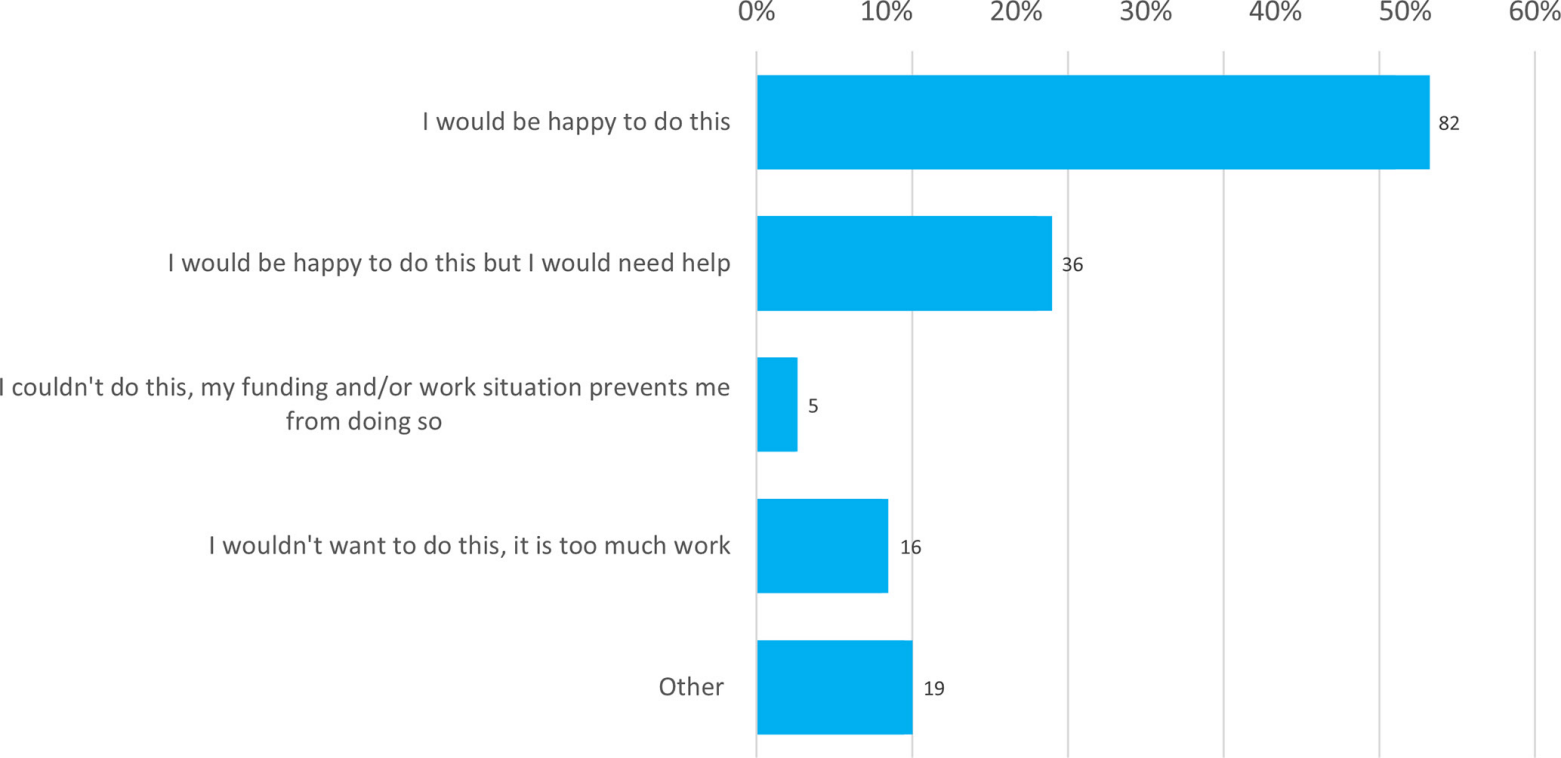
Willingness of respondents to make their underlying data open. Answers shown were given in response to the question ‘The open research platform aims to ensure that, where possible, all raw data underlying the results is deposited and made available, so that others can analyse, replicate and reuse your work. As an author, how would you feel about being asked to make all of the data associated with your article open? This would include, as examples, all sequencing data and/or software source code.’ Total responses are indicated above each bar.

75 % of respondents said that they would be happy to make the data underlying their research open, whether needing assistance to do this (23 %, *n=*36) or not (52 %, *n=*82; Fig. 3). 13 % of respondents indicated either they did not want to (10 %, *n=*16) or they would not be able to because of their work or funding situation (3 %, *n=*5). Of those who selected ‘Other’ (12 %, *n=*19), most of these (*n=*11) indicated an agreement with the policy in principle or a willingness to do so, but cited possible barriers to making the data open. These included: the ease of doing so; funding or work restrictions; copyright and patent issues; and patient confidentiality.

### Types of research

**Fig. 4.**
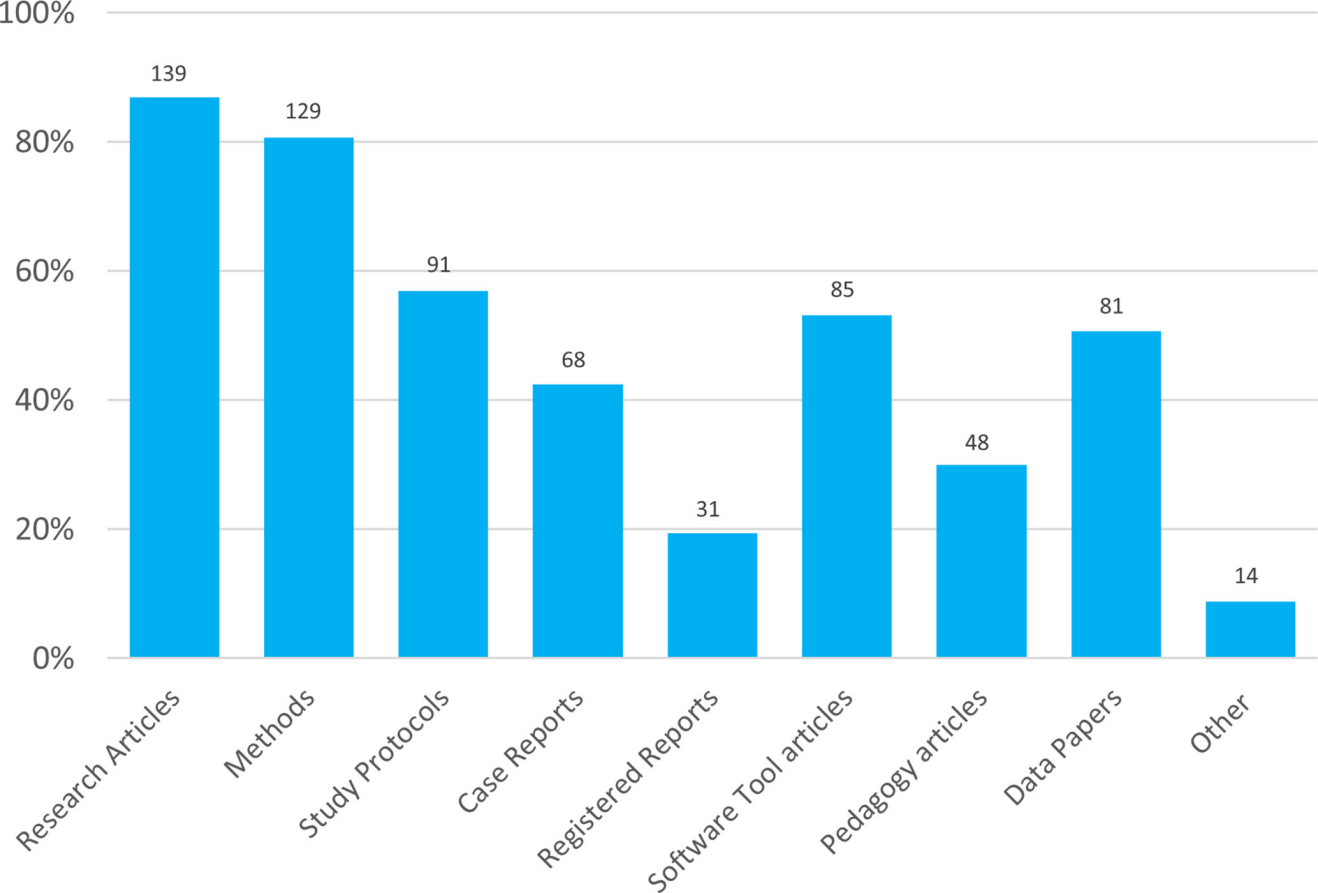
Types of research that respondents would like the open research platform to publish. Answers shown were given in response to the question, ‘What type of research would you like the open research platform to publish? (Please select all that apply)’. Total responses are indicated above each bar.

There was a level of interest in all the article types listed in the survey, ranging from 89 % of respondents indicating they wanted to see Research Articles published on the platform, to 20 % for Registered Reports (Fig. 4). A few who selected ‘Other’ indicated a desire for Reviews, whilst a couple suggested replication or negative data, which would be published as Research Articles or Short Communications. Another respondent suggested Outreach papers, which could be published as Pedagogy articles. It is therefore clear that there is some confusion about what research can be published under what article type.

### Submission history to *Access Microbiology*

**Fig. 5.**
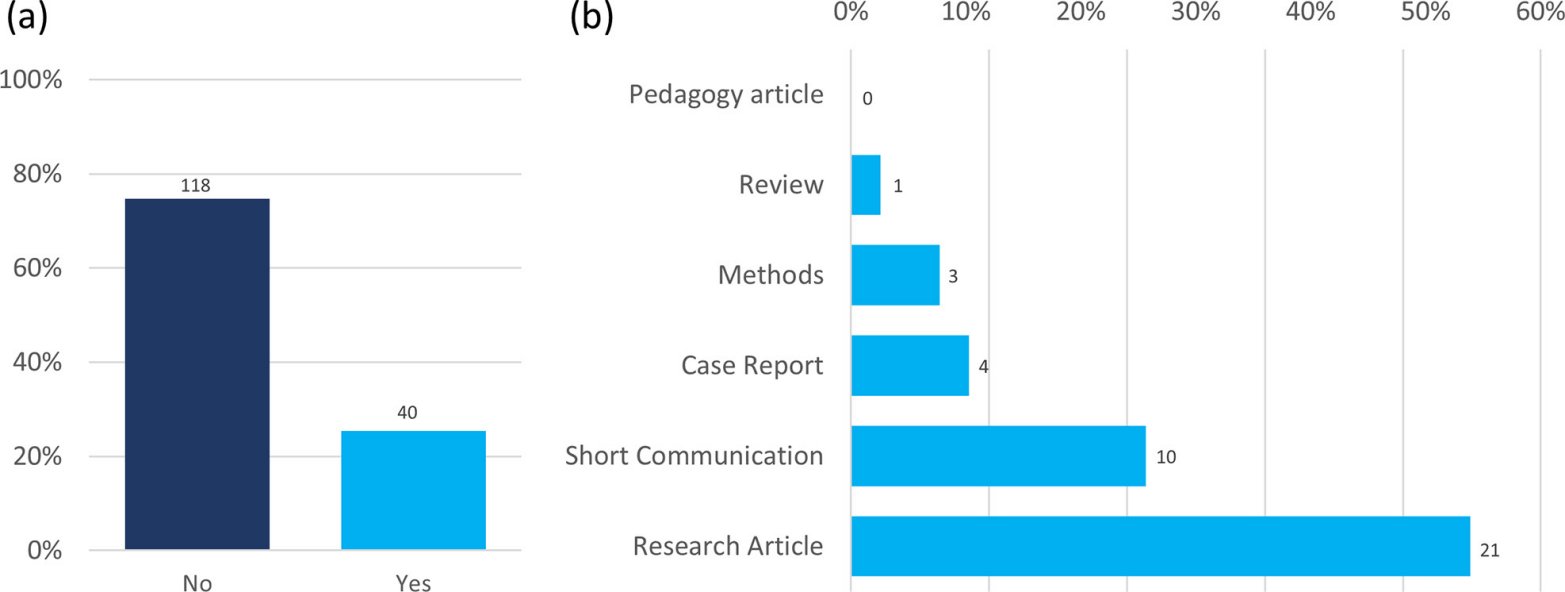
Submission history to *Access Microbiology*. (**a**) Answers shown were given in response to the question, ‘Have you previously submitted to *Access Microbiology*?’. (**b**) For those who answered ‘Yes’ to ‘Have you previously submitted to *Access Microbiology*?’, ranswers shown were given in response to the follow-up question, ‘What type of article did you submit your work as?’. Total responses are indicated above each bar.

25 % of respondents stated they had previously submitted work to *Access Microbiology* (Fig. 5a), and the majority of that work was submitted as a Research Article (54 %, *n=*21) or Short Communication (26 %, *n=*10; Fig. 5b).

### Previous submission to open research platforms and likelihood of submitting to *Access Microbiology*

**Fig. 6.**
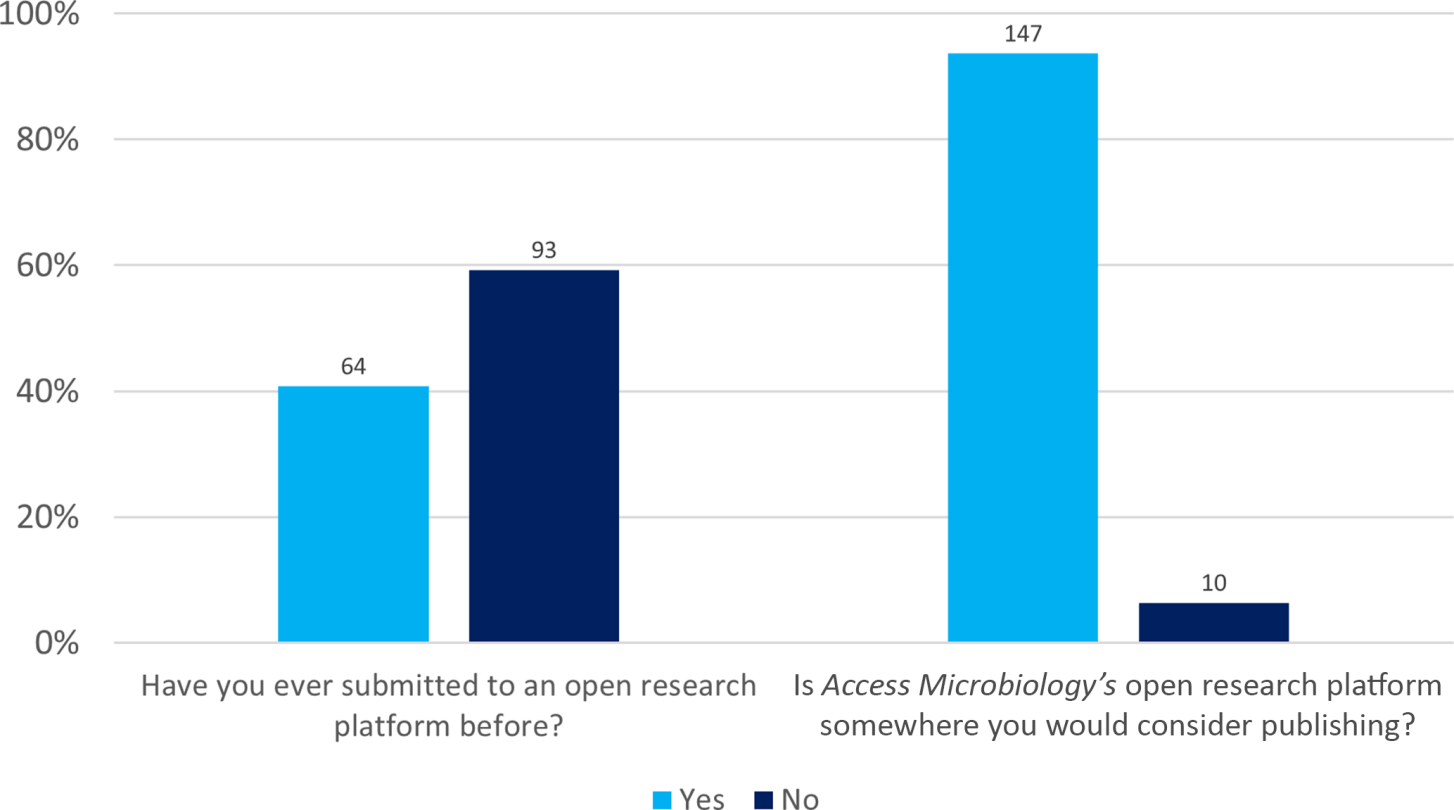
Previous submission to open research platforms and the likelihood of submitting to the *Access Microbiology* open research platform. Total responses are indicated above each bar.

Whilst only 41 % of respondents stated they had previously submitted to an open research platform, 94 % stated that the *Access Microbiology* open research platform is somewhere they would consider publishing (Fig. 6).

The few respondents who said they would not submit were Early Career researchers (*n=*4, 11 % of that group), those in Established careers (*n=3*, 6 % of that group), Mid-career (*n=*2, 5 % of that group) and Other (1). Reasons included: not having sufficient funds (2); too little impact to warrant submission (2); not having a high Impact Factor; dislike of the use of artificial intelligence tools; preferring to post-separately on preprint servers and not commit to a journal; and not having enough information at present to decide.

### Career stage

**Fig. 7.**
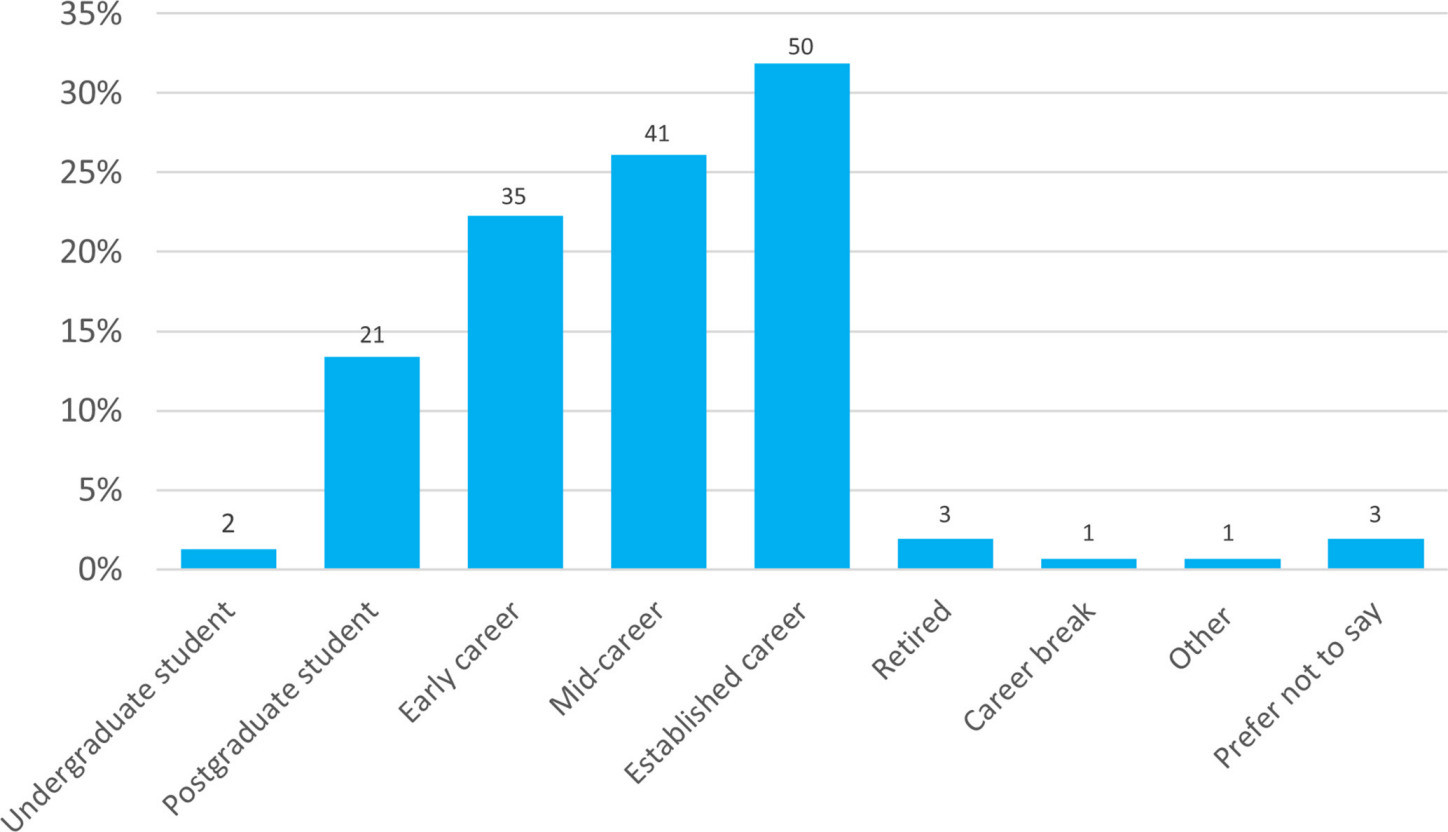
Career stage of respondents. Answers shown were given in response to the question, ‘What career stage are you currently at?’. Total responses are indicated above each bar.

There was a broad representation of all active career stages, with those identifying as being in as Established career representing the highest proportion at 32 %, and Mid-career (26 %), Early career (22 %) and Postgraduate students (13 %) following (Fig. 7). Making up the last 6 % were: Retired; Preferred not to say; Undergraduate students; Career break; and Other.

### Area of work

**Fig. 8.**
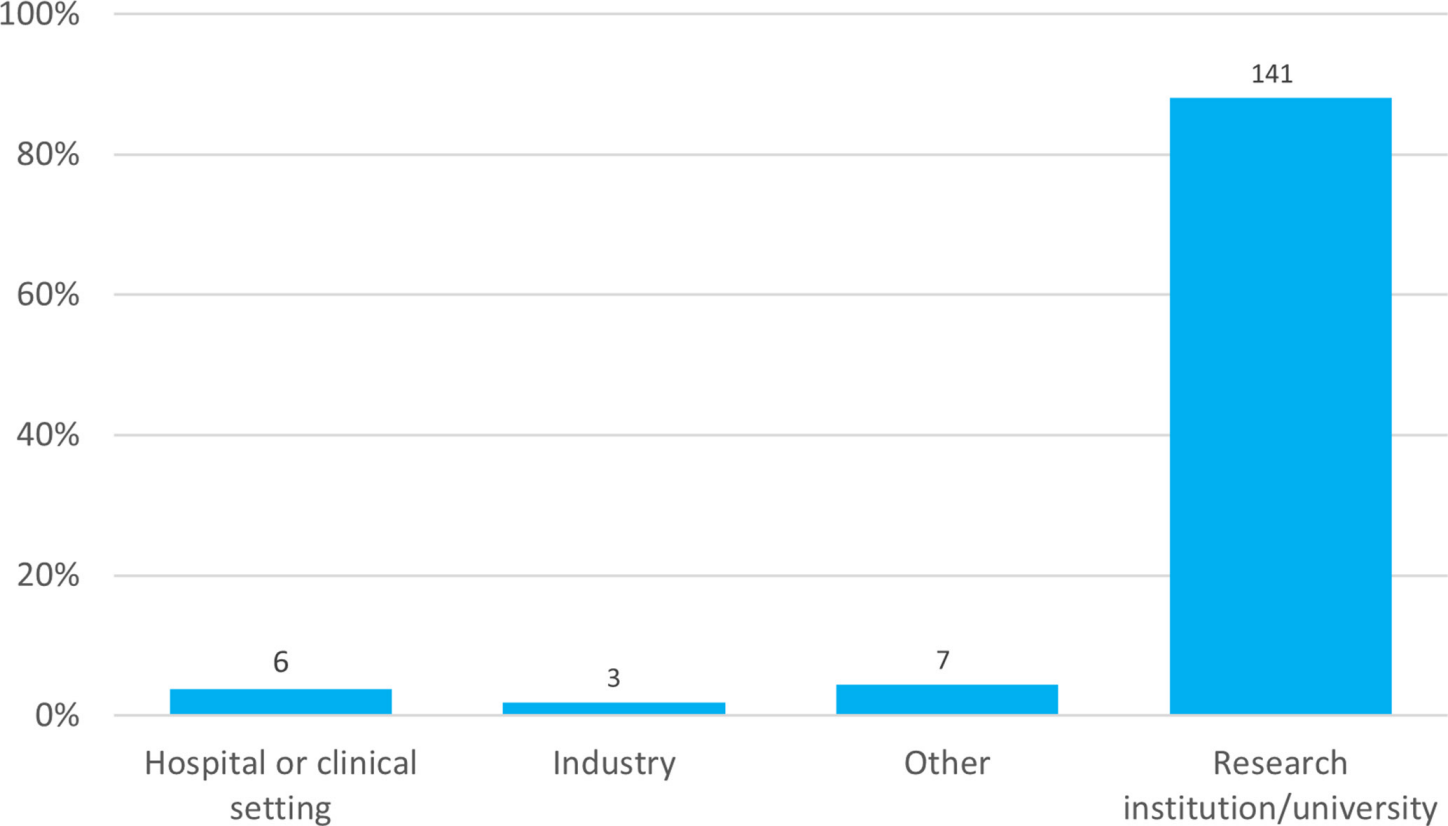
Area of work. Answers shown were given in response to the question, ‘What area of work do you work in?’. Total responses are indicated above each bar.

The vast majority of respondents (90 %) work in a research institution or university (Fig. 8). Sharing medical or clinical research prior to full peer review as preprints is often cited as a concern of researchers in those fields due to the possible societal risks and implications, although there is limited research on this. With such a small proportion stating they work in hospitals or a clinical setting (4 %, *n=*6) it was therefore not possible to assess the relationship between these types of researchers and likelihood of submitting to the *Access Microbiology* platform.

### Selected comments from respondents

#### Positive comments

- ‘I like the idea of publishing the full review alongside the paper.’
- ‘Very glad to see this society trying to embrace modern scientific publishing, unlike some which seem to see it as a revenue stream and actively fighting open access etc.’
- ‘Steps for Microbiology Society and its publications to have more visibility.’
- ‘The idea is really good, AI will make review process easier.’
- ‘I think the direction the journal is taking the wonderful [sic]. As an author, this would make me more keen to submit to AM, but as a reviewer, I might be more likely to decline invites to review knowing my name would be associated with my comments.’
- ‘The concept of a DOI-citable microbiology-specific pre-print platform is very appealing.’

#### Negative comments

- ‘Avoid being just a dumping ground for papers rejected by other journals!’
- ‘Refocus on the flagship journal and stop chasing fads. I agree with the ideals the new journal wants to achieve but better, well established alternatives already exist.’
- ‘I reckon you need to be clearer about the vision.’

#### General comments

- ‘Please consider special rate or free open access to countries of low income. Researchers work hard with limited budget and again face difficulty while publishing. This is not fair.’
- ‘Is there a way of getting direct updates for certain specialties? For example, antimicrobial resistance… like a filter tool?’
- “It would be great to create major areas and their respective subareas where the submitted manuscripts could be assessed by the community. For instance, ‘Environmental Microbiology/Microbial Ecology’ with the subarea ‘Marine Microbiology’.”
- ‘The platform should be user-friendly.’
- ‘Make sure you clarify the term ‘open data’ -- slippery ground.’
- ‘The cost of the papers shouldn't be a problem for research teams that have low funding (including in industrialized countries).’
- ‘Don't let the quality of the publications suffer in an effort to get people to engage.’

## Conclusions and recommendations

The fact that 59% of respondents have never published on an open research platform (Fig. 6a), yet 94 % indicated that the platform is somewhere they would consider publishing is a convincing response and shows clear enthusiasm and interest in the platform from those who completed the survey. However, it is important not to conflate ‘considering submission’ with ‘intention to submit’. In addition, it should not be assumed that the survey represents the views of the broader microbiology community as a whole, as surveys have intrinsic self-selection bias and may not reflect the wider community.

A very high proportion of respondents (75 %) said that they would be happy to make the data underlying their research open, whether requiring assistance to do this or not, whilst 13 % could not or would not want to. Many of the reasons provided in ‘Other’ referred to funding or work restrictions, copyright and patent issues, and patient confidentiality. The H2020 Programme Guidelines on FAIR Data recognises that there are ‘good reasons to keep some or even all research data generated in a project closed’ and recommend that data should be ‘as open as possible and as closed as necessary’ [4]. Many of these reasons would fall under this exemption if we were to adopt this principle, and so there is clear scope and enthusiasm from the community surveyed to adopt this principle. However, with over a third of respondents indicating they would need help to do this, any implementation of this policy would need to be coupled with clear guidance on our ‘Information for Authors’ pages, including what open data means, our recommended repositories, best practices, and support to help authors directly deposit their data in the Society’s Figshare portal.

With such a high proportion of respondents indicating they would always or sometimes want to remain anonymous on their peer review report, imposing an open peer review model could alienate many potential reviewers. Even with the slightly surprising result that respondents across career stages had a similar willingness to provide names to reviews, there are evidently still genuine concerns about the repercussions of performing a critical review of a senior researcher or colleague. Difficulty securing reviewers is a publishing-industry-wide problem [5] and *Access Microbiology* is no different. The risk of dramatically decreasing the reviewer pool and potentially increasing pressure on those who would continue to perform reviews is a risk to the platform’s sustainability.

Given the interest in a wide range of article types, this suggests that those surveyed are knowledgeable on the importance of sound-science research and are eager to have a place to publish it. A unique workflow for Registered Reports will need to be established, so inclusion of this in the first launch phase should be considered. The suggestions of sound-science research (e.g. replication or negative data) or Outreach papers in the ‘Other response’ section suggests that there is confusion amongst some of the community as to what article types can be used for what research type, so this should be considered prior to launch.

Finally, the open-ended feedback question provides additional insight into participants perspectives or concerns about the platform. There were numerous positive responses, showing many respondents are supportive of what the Microbiology Society is undertaking. However, there was a concern from multiple respondents regarding the cost of publishing. It will be completely free for authors to submit and publish for the first 12 months of the platform launch – this is because the Wellcome grant is funding the first year of running costs, so we can subsidize all costs for authors. However, once article processing charges (APCs) are introduced, the Society waives all open access fees for corresponding authors from both A and B Hinari countries, as well as providing fee-free open access through Publish and Read agreements. Therefore this free-to-publish launch period and our other policies must be communicated clearly to authors considering submitting to the open research platform.

**Recommendations:**

1. **Adopt a transparent peer review model**. Provide explanation on our website of the advantages of this model and how it works on our platform. Consider surveying reviewers who decline to provide their name to their review to understand their concerns and attempt to address these.
2. **Adopt an open data policy, with an underlying principle of ‘as open as possible, as closed as necessary’**. Provide a clear explanation on our website of why we have adopted this policy, what it means and why it benefits authors and the wider community. Provide guidance on the website on best practices, recommended repositories and help in depositing their data in our Figshare portal.
3. **Launch with most article types listed in the survey, but investigate possibility of including Registered Reports**. Provide clear guidance on our website and in communications about which article types encompass the various types of research output, and to encourage authors to contact us if unsure.
4. **Communicate that it will be free to publish for the first 12-months after launch and eligibility for fee-free open access publishing to those authors who qualify**. Provide clear guidance on our website and in communications to encourage those authors to submit.

**Survey questions**

This box provides a full list of the questions present in the survey. If applicable, multiple-choice answers have been given as bullet points below the question.

  1. To increase transparency during the peer review process, the platform will publish peer review reports for each version of an article, preferably with the reviewer name(s) alongside. Knowing this, how would you feel about providing your name to your review?

    •I would always be happy for my name to be published with my review.

    •I would be happy for my name to be published sometimes but there are times I would want to remain anonymous.

    •I would always want to remain anonymous and would decline to review if it is mandatory to include my name.

  2. What would the reasons be for your answer? (I would be happy for my name to be published sometimes but there are times I would want to remain anonymous.)

  3. What would the reasons be for your answer? (I would always want to remain anonymous and would decline to review if it is mandatory to include my name.)

  4. The open research platform aims to ensure that, where possible, all raw data underlying the results is deposited and made available, so that others can analyse, replicate and reuse your work. As an author, how would you feel about being asked to make all of the data associated with your article open? This would include, as examples, all sequencing data and/or software source code.

    • I would be happy to do this. I would be happy to do this but I would need help.

    • I wouldn’t want to do this, it is too much work.

    • I couldn’t do this, my funding and/or work situation prevents me from doing so.

    • Other (please state).

  5. What type of research would you like the open research platform to publish? (please select all that apply)

    • Research Articles

    • Methods

    • Study Protocols

    • Case Reports

    • Registered Reports

    • Software Tool Article

    • Pedagogy articles

    • Data papers

    •Other (please state)

  6. Have you previously submitted to *Access Microbiology*?

    •Yes

    •No

  7. What type of article did you submit your work as?

    •Research Article

    •Short Communication

    •Methods

    •Case Report

    •Review

    •Pedagogy

  8. Have you ever submitted to an open research platform before?

    •Yes

    •No

  9. Is *Access Microbiology’s* open research platform somewhere you would consider publishing?

    •Yes

    •No

    •If no, please explain the main reason(s) why you wouldn’t consider publishing

  10. What career stage are you currently at?

    •Career break

    •Undergraduate student

    •Postgraduate student

    •Early career

    •Mid-career

    •Established career

    •Retired

    •Prefer not to say

    •Other (please describe)

  11. What area of work do you work in?

    •Research institution/university

    •Industry

    •Hospital or clinical setting

    •Other (please state)

12. Do you have any other feedback or suggestions about *Access Microbiology’s* open research platform?
